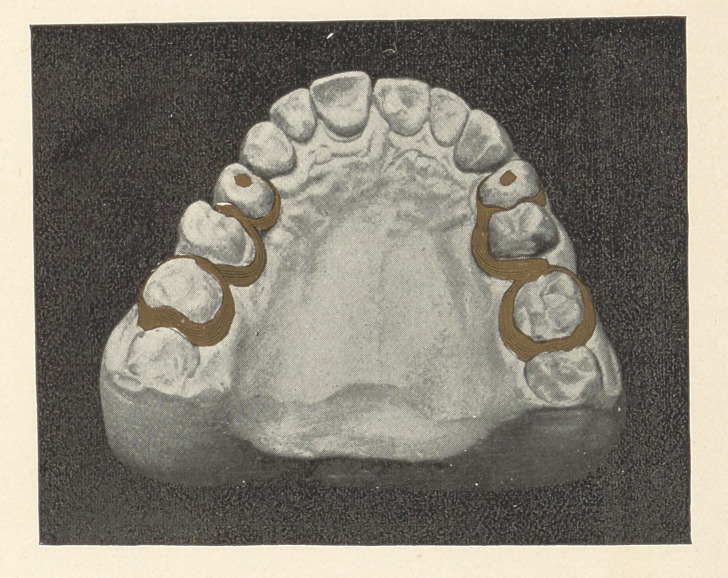# The Meriam Extension Crown

**Published:** 1899-03

**Authors:** Horatio C. Meriam

**Affiliations:** Salem, Mass.


### International Dental Journal.

Vol. XX. March, 1899. No. 3.

### Original Communications.[1]

### THE MERIAM EXTENSION CROWN.[2]

BY DR. HORATIO C. MERIAM, SALEM, MASS.

Some years since, I wished to insert a first superior bicuspid without a plate, but objected to cutting into the second bicuspid or the cuspid. I neither wished for the display of gold that would come from banding these teeth, nor did I think well of bands or pins built into teeth that were to be subject to strain in mastication. A new device seemed to be called for, and my plan resulted in the method that I am now to report.

The first molar had been lost and the teeth had separated; the root of the first bicuspid was in place, but was decayed very much and so far up in the alveolus that crowning was impossible. I wished to arrange for sightliness, cleanliness, stability, and utility, together with ease of repair, if need be, and yet to avoid injuring, marring, or trimming the other teeth.

You will recall that the second superior molar often tapers slightly, being a little larger at the neck than at the crown, and

[1] The editor and publishers are not responsible for the views of authors of papers published in this department, nor for any claim to novelty, or otherwise, that may be made by them. No papers will be received for this department that have appeared in any other journal published in the country.

[2] Read before the American Academy of Dental Science, October 5, 1898.

so will allow a band to be fitted and forced up tightly without trimming. This tooth was banded, the band being made extra thick on the buccal surface, so that it could be cut narrow and show a portion of this surface of the tooth. The remaining portion of the root of the bicuspid was cleaned and filled with gutta-percha, and a small oval cup of heavy plate was made to fit against it, opening downward. An English tube-tooth was selected, ground to place, and then banded, the band being left wide on the palatal surface. This band was fitted and soldered to the cup, which was then drilled through, corresponding to the hole in the tube-tooth. A platinum-iridium post was soldered in place. This post was long enough to come nearly through the tooth, and was slotted to retain a gold filling (which was inserted after finally setting the tooth in the cap with gutta-percha). The cap was connected with the band on the molar by a strong bar of spring gold wire, No. 16 or 17, or larger, English standard gauge, curved around the bicuspid, leaving it free and clean. An impression was taken with the molar band in place; the cast was then made with powdered pumice mixed in the plaster, the cap and the bar were placed in position, and the piece was then invested and soldered together. In subsequent cases I have either bent up the bar a little where it touched the cap, or have let it run under the cap a little where the alveolus has been absorbed; or, if there was room, have let it pass along the distal surface of the cap. After soldering I have cut the cap away on the labial surface to a mere line, leaving it wide on the palatal and distal surfaces. The cap may be cut narrow or left full width on the mesial surface, the cutting being governed by the amount of gold in view. The tooth is then put in place with gutta-percha or cement, the gold filling inserted and polished, and the piece set in place. The case here, that shows two of these crowns *in situ*, will make it clear to you all.

I have described the one resting against the root. The other, you notice, shows a slight absorption of the alveolus.

You will recall that the law requires (I quote from memory) that a new device shall be accompanied by "such a description as will enable one skilled in the art to which it appertains to make a duplicate." I feel that gentlemen of the standing of the Fellows of the Academy will have no trouble in exceeding the little skill I have in designing and making what I have shown and here present. (See illustration.)

I wish to call attention to one point,—viz., that a tooth with a band secured to a tooth beyond those standing beside the vacant space has the advantage that the tooth is less likely to be tipped, having the full support of the alveolus, and that the alveolus is less likely to be absorbed.

### EXTRA TOUGH GOLD SOLDER.

It is desirable to avoid uncertain formulæ in the making of plate or solder. We used to see formulæ that called for the addition of a certain proportion of brass wire or brass pins. Now, brass and brass wire vary, and we can work best if we start with each metal pure and distinct. A very small portion of some metals will make gold intractable. I remember that I once took some gold—old fillings—to be made into pure gold wire, and my man complained that it worked "short." The action of metals in contact in a state of fine division is an interesting one, but too long for this report; but when we learn that one part of tin in a thousand will make gold too brittle to work, we can understand the importance of what I allude to. We use tin constantly in filling, either by itself or in combination with gold, and I think that none of us would overlook this source of danger in making plate or solder, but I think we have not thought of the tin in amalgam; a very small part, even that in an amalgam repair of a gold filling, might ruin the working of plate or solder. I direct my maker to treat all my gold with corrosive sublimate for the removal of tin. The pennyweight is taken as a starting-point and divided into this formula:

| | Gold. | Silver. | Copper. | Zinc. | |
|---|---|---|---|---|---|
| Plate . . . . . . . . . . . | 18 gr. | 5 gr. | 1 gr. | 0 | = 24 gr. |
| Solder . . . . . . . . . . . | 18 gr. | 3½ gr. | 1 gr. | 2 gr. | = 24½ gr. |

You see by this that a portion of the silver is withheld and zinc added to make the melting-point of the solder lower than that of the plate, and that there can be but slight difference in color between them. The slight quantity of copper helps to toughen the solder, and deepens the color of the plate; a lighter-colored plate and a very free flowing solder are made by omitting the copper and adding an equal amount of silver. But the underlying rule of pure metals, and lowering the melting-point of the solder by withholding part of one metal and adding an equal part of another of the same color but of a low melting-point, such as zinc, will allow us to make a plate and solder of any carat that will work well together. You perhaps noticed that the parts given for the solder foot up twenty-four and one-half grains; an excess of zinc is added to allow for loss in melting and in soldering.

In the work that I show you the bands are made of this plate, reinforced and soldered with solder made by this formula. One side is not polished, and you can see where the solder has flowed; the polished side shows the colors of the plate and solder and the excellence of the match. You will notice the pieces of plate and solder that are now being passed around, and can judge of the color and test the toughness of the solder. You will see that the solder is rolled very thin. I like this for convenience in use and that it cannot be mistaken for plate in the office. I have twenty pennyweights made up at a time, and if of the same thickness they might get mixed.

### ANNEALING STEEL.

Man has worked steel from very early times. As boys we were told stories of wonderful blades, the secret of whose making perhaps died with the makers. The blades of some of these old makers are now highly prized by those who make collections.

We have been accustomed to think of steel depending for its quality on its tempering, and by tempering I mean heating to redness, hardening, and then drawing the temper to that desired, either for cutting, springs, or other purposes; but piano wire shows us that a certain quality of steel is produced by drawing, so that it is practically fibrous. The sword-maker's skill was shown in the forging, and he often worked the blade while cold until he got the right temper into it. Jewellers make a small drill, without heat, from pivot wire (piano wire) by flattening it slightly on an anvil, but they do not turn it and strike the sides, for that would break the arrangement of the fibres and cause the drill to split. Many flat instruments can be made in this way if the forging is confined to the side.

Steel for the ordinary instrument is completely annealed in a charcoal fire, and is not brought to the air until the fire has burnt down and the steel is completely cold, thus avoiding even the slight hardening of the surface that might come from contact with the cool air, and giving a uniform annealing. Hardening and annealing are now believed to represent different arrangements of the molecules. This point is not very clear: the supposition remains that the chemical composition is unchanged.

These general facts help us to understand some of our failures in working steel. In drawing the temper of an instrument in a gas flame or a fire and bringing it to the air we get a partial cooling of its surface, so that it is not homogeneous and is unlike the steel annealed in a charcoal fire. In making a small drill, workmen, after forming it, heat it to redness and swing it quickly through the air, and find this hardens it sufficiently.

Spatulas, scalers, and plastic filling-instruments can be repointed by heating to redness in a flame, bending while there into the required shape, and then flattening on an anvil. Such an instrument will be found hard enough for its work, but soft enough to file to shape or change a little as required while operating. The scaler of Dr. Lord's pattern can easily be made in this way, and I show burnishers made thin and broad, for burnishing contour fillings between the back teeth with a slight rotary motion. They are soft enough to bend a little, but you can test the temper, which is all given by striking on an anvil when cold. They have not been in the fire since bending in the flame. You will notice the appearance of the blade, which is different from that of blades filed to thinness. Some of the instruments shown are repointed from steel that is unsuited for anything but scalers, but these were made in the same way. On some of them you will see serrations such as Dr. Lord now has put on his instruments. Some also are serrated on the back; one is made long and thin, with serrations on each side, and flexible enough to bend and pass between the back teeth at the cervical wall. These are quickly made from excavators, and if one breaks it is only a moment's work to make another. The serrations are made by Dr. Lord with a "crossing file," which I show you.

I now come to a matter which I wish more especially to report, as I do not know that it has ever been brought up in dentistry, nor do I find it in any work that has come under my notice. I alluded a moment ago to the hardening of steel by sudden cooling, the hardening of a small drill in the air, and the partial hardening by being brought suddenly to the air before cooling. It can, therefore, be seen that if we wish to draw the temper of a small, hard instrument, and should draw it, in the usual way, to the color wished for, and then plunge it into water or oil, while we should get the usual result, that result might not be a tough, homogeneous piece of steel, but a piece whose outer surface was harder, or different from the inner, and which was consequently more liable to break. I show here a lot of the small sizes of milliners' needles, which are very slender and about the length of broaches. These have been annealed, and you can see that they can now be bent and twisted in any direction, almost tied in a knot and straightened again, and all the while showing a spring temper. They are soft enough to file and can be made very thin,—a few days since I was able to follow the pulp-canal in a bicuspid in the mouth of a lady nearly seventy years old.

The needles were, of course, hard when I took them, and were placed, point down, in a thin metal-screw-top bottle, which had a few small holes bored in the top to allow for the expansion of air. The bottle was then grasped at the top with a small pair of soldering tongs, bent to grasp it firmly, and passed, bottom down, back and forth over a bunsen flame until I saw the color come that I wished. The bottle was then withdrawn from the flame and allowed to cool. It could cool but slowly, as glass is a poor conductor and the holes admitted but a small amount of air. In my next lot I shall use a long test-tube, corked, with a slit cut along the side of the cork to allow for expansion and to limit the admission of air. This method may prove of little value, but I am glad to have a means of attaining a uniform temper in so small an instrument. I have not tried to barb them, and doubt if it can be done for the smaller sizes.

In closing let me show two plugger-points that I had made for me by Mr. Grafrath. They are curved right and left, but the points are brought into line with the blow of the mallet.

## Figures and Tables

**Figure f1:**